# Co-existence of other copy number variations with 22q11.2 deletion or duplication: a modifier for variable phenotypes of the syndrome?

**DOI:** 10.1186/1755-8166-5-18

**Published:** 2012-04-09

**Authors:** Deling Li, Mustafa Tekin, Maria Buch, Yao-Shan Fan

**Affiliations:** 1 Department of Pathology, University of Miami Miller School of Medicine, Miami, FL 33136, USA; 2 Dr. John T. Macdonald Department of Human Genetics, University of Miami Miller School of Medicine, Miami, FL 33136, USA; 3 John P. Hussman Institute for Human Genomics, University of Miami Miller School of Medicine, Miami, FL 33136, USA; 4 Jackson Memorial Hospital, University of Miami Miller School of Medicine, Miami, FL 33136, USA

**Keywords:** DiGeorge syndrome, 22q11.2 microdeletion, 22q11.2 microduplication, Array CGH, Copy number variations (CNVs)

## Abstract

**Background:**

The phenotype in patients with a 22q11.2 deletion or duplication can be extremely variable, and the causes of such as variations are not well known.

**Results:**

We observed additional copy number variations (CNVs) in 2 of 15 cases with a 22q11.2 deletion or duplication. Both cases were newborn babies referred for severe congenital heart defects. The first case had a deletion with a size of approximately 1.56 Mb involving multiple genes including *STS *in the Xp22.31 region along with a 22q11.2 deletion. The second case had a duplication of 605 kb in the 15q13.3 region encompassing *CHRNA7 *and a deletion of 209 kb involving the *RBFOX1 *gene in the 16p13.2 region, in addition to 22q11.2 duplication.

**Discussion:**

Our observations have shown that additional CNVs are not rare (2/15, 13%) in patients with a 22q11.2 deletion or duplication. We speculate that these CNVs may contribute to phenotype variations of 22q11.2 microdeletion/duplication syndromes as genomic modifiers.

## Background

It is well known that the phenotype of patients with 22q11.2 microdeletion or microduplication can be extremely variable from near normal to severe developmental disabilities including congenital heart defects, learning disabilities, and risk of psychological problems such as attention-deficit hyperactivity disorder, autism-spectrum disorders and schizophrenia [1,2]. The mechanisms that lead to extreme phenotype variations in this syndrome remain unknown. Prior to the clinical use of microarray based comparative genomic hybridization (array CGH), vast majority of patients with a 22q11.2 deletion or duplication were diagnosed by FISH [3-5], and therefore genomic changes other than the 22q11.2 region were barely known. Here, we report additional genomic aberrations detected by array CGH in two newborns, one had a deletion and the other had a duplication of the 22q11.2 region.

## Case presentation

### Patient 1

The patient was an infant girl, 15 days of age, born to a 23 year old healthy mother. The baby was delivered at the 38th week of gestation by C-section due to nuchal cord. The baby weighted 2.9 kg, with Apgar scores 8 at 1 min, 9 at 5 min, and 9 at 10 min. Management of the infant at delivery included stimulation and suction. Echocardiography showed tetralogy of Fallot with absent pulmonary valve and left hemitruncus. A computerized tomography (CT) scan of the thorax with contrast and 3D reconstruction showed a large right pulmonary artery compressing on both right and left main stem bronchi, but more significantly on the right bronchi during systole. The infant also had mild respiratory distress at birth, anemia, and feeding intolerance. She became increasingly tachypneic with chest X-ray findings of air trapping on the right lung, pulmonary edema on the left lung, and cardiomegaly. Abdominal ultrasound showed bilateral small kidneys. Other physical and ultrasound examinations were unremarkable, except for overriding toes.

### Patient 2

Patient 2 was an infant boy, 15 days of age. He was delivered at 37th week of gestation to a 19-year-old mother by C-section due to IUGR. His birth weight was 2.25 kg with Apgar scores of 9, 9 and 9. No complications were reported in the mother during this pregnancy. The baby had normal tone and reflexes with apparently normal activities. He had micrognathia without other specific dysmorphic features. Systolic ejection murmur at the lower sternal border was noted. Ultrasound showed complex congenital heart defects including double outlet right ventricle with a large ventricular septal defect and pulmonary stenosis, mild right ventricular hypertrophy, mild tricuspid valve hypoplasia, pulmonary valve hypoplasia, and patent foramen ovale with bidirectional shunting. Other obvious abnormalities were not noted by physical examinations and routine laboratory tests. The maternal side of the family had a history of controlled hypertension and heart murmur without complications while the paternal side history was unremarkable.

## Results

**Figure 1:**
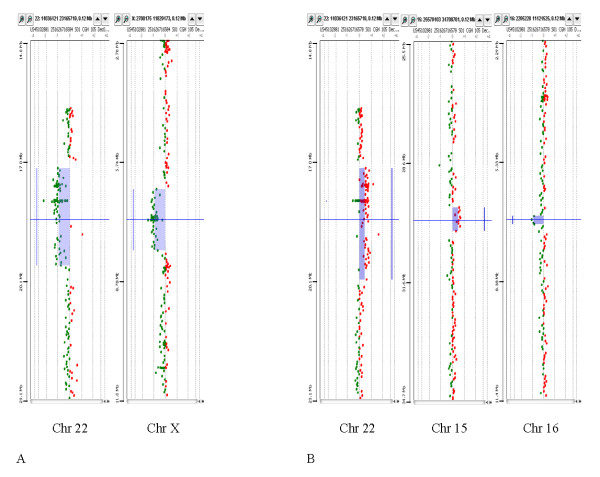
**Complex pathogenic imbalances in two patients**. A. A 2.47 Mb deletion at 22q11.21 and a 1.56 Mb deletion at Xp22.31 in patient 1. B. A 2.84 Mb duplication at 22q11.21, a 0.61 Mb duplication at 15q13.3, and a 0.21 Mb deletion at 16p13.2 in patient 2.

In patient 1, array CGH showed a 2.47 Mb deletion in the 22q11.2 region and a 1.56 Mb deletion in the Xp22.31 region (Figure 1A). In patient 2, array CGH detected multiple CNVs, including a 2.84 Mb duplication in the DiGeorge region, a 605 kb duplication in the 15q13.3 region and a 209 kb deletion in the 16p13.2 region (Figure 1B). Details of the CNVs in the two patients are summarized in Table 1. Conventional chromosome analysis revealed a normal karyotype in both patients. Parental studies were offered to the parents of both proband patients but were declined.

**Table 1:** CNVs involved in the two patients (hg18)

Patient	Imbalance	Size _(MB)_	ISCN description	Genes involved
1	del	2.47	22q11.21(17299942-19770514) × 1	More than 40 genes including TBX1
	del	1.56	Xp22.31(6498721-8057511) × 1	HDHD1A, STS, VCX, PNPLA4
2	dup	2.84	22q11.21(17299942-20139009) × 3	More than 40 genes including TBX1
	dup	0.605	15q13.3(29818304-30423251) × 3	CHRNA7
	del	0.209	16p13.2(6776911-6986220) × 1	RBFOX1

## Discussion

22q11.2 microdeletion and microduplication syndromes are mediated by nonallelic homologous recombination (NAHR) between region-specific low-copy repeats (LCRs) during meiosis [6]. The number of patients with a microduplication appears to be smaller than that of patients with a deletion although an equal frequency is expected [7,8]. We have detected microdeletion or microduplication of the 22q11.2 DiGeorge region in 15 of 1292 cases (1.16%) referred for intellectual disabilities and/or congenital anomalies in the past several years. Among them, ten patients had a 2.4 Mb ~ 2.9 Mb deletion; two patients had a duplication of the same region; one had a 1.4 Mb deletion and two had a 500-600 Kb duplication.

A DNA segment with a size of approximately 3 Mb that encompasses over 40 functional genes is involved in about 90% of 22q11.2 microdeletions or microduplications [2]. Clinical features of congenital heart defects in 22q11.2 deletion syndromes are usually associated with the common 3 Mb deletions involving *TBX1*, while atypical deletions may show variable phenotypes modified by deletion location, size and other factors [9,10]. However, extremely variable phenotypes have been reported in patients with the same 22q11.2 deletion, and the mechanisms leading to such variations remain unknown [11].

We detected complex CNVs in 2 of the 15 cases (13%), suggesting that additional CNVs are not rare among the patients with a 22q11.2 microdeletion or microduplication. Our patient 1 had an Xp22.31 deletion involving *HDHD1A, STS, VCX, PNPLA4 *genes in addition to the typical 22q11.2 deletion. STS deletion is associated with X-linked ichthyosis (XLI) in male due to deficiency of steroid sulfatase (*STS*) activity [12]. We speculate that the genes involved in Xp22.3 and 22q11.2 deletions may interact in normal development given the large number of genes involved.

Our patient 2 had a duplication of the *CHRNA7 *gene on chromosome 15q and a partial deletion of the *RBFOX1 *gene on chromosome 16p in addition to the 22q11.2 duplication. The microduplication phenotypes can be very different even within the same family with the exactly same duplication [6]. While the majority of patients with a 22q11.2 deletion have congenital heart defects, most of the cases with duplication of this region do not have [6]. Our patient 2 had severe congenital heart defect. It is not known if the additional CNVs have contributed to the severe abnormal heart development in this patient, but this possibility cannot be excluded. It is well known that neuropsychiatric disorders such as schizophrenia, attention deficit disorder, depression and autism frequently occur in the individuals with a 22q11.2 deletion or duplication [13-15]. Copy number variations of *CHRNA7 *and *RBFOX1 *have been reported in patients with mental retardation, autism and schizophrenia or seizure [16-18]. It is likely that CNVs of *CHRNA7 *and *RBFOX1 *would add additional risk for psychological diseases in the patient when he grows up.

## Conclusions

In summary, the observation of additional CNVs in our two cases with 22q11.2 microdeletion/duplication suggests that complex genomic changes are not rare in DiGeorge patients and they may, as genomic modifiers, contribute to the variable phenotypes of the disease. Continuing follow-up with these two patients and accumulation of more cases similar to ours in literature may help our understanding on the complicated phenotypes of the 22q11.2 microdeletion/duplication syndrome.

## Materials and methods

### Chromosome analysis

Conventional GTG banded chromosome analysis was performed at the level of resolution of 500-550 bands per hypoid and reported according to ISCN 2009.

### Array CGH

Array CGH was performed as described [19]. Briefly, DNA was extracted from patient peripheral blood using commercially available DNA isolation kits (Qiagen Sciences, Maryland) according to the manufacturer's instructions. A customized genome-wide oligonucleotide array (Agilent Technologies, Santa Clara, CA) was used for the studies. This platform was composed of 44,290 60-mer oligonucleotide probes with an average spacing of 30-35 kb genome-wide. Probes are enriched for the regions and genes known to be associated with syndromes or developmental disorders. Patient and reference DNA samples were differentially labeled and cohybridized to the arrays. Hybridized slides were scanned with microarray scanner (Agilent G2505B), and analyzed with Feature Extraction and DNA Analytics 4.0 (Agilent Technologies). The assembly hg18/build36 of the human genome was used for the two cases.

## Consent

All patients under the care of Jackson Memorial Hospital (JMH) signed a written informed consent that allows the medical staff of JMH and University of Miami School of Medicine to use their medical records for medical research and education purpose without revealing patient identification. A copy of the written consent is available to review by the Editor-in-Chief of this journal.

## Abbreviations

FISH: fluorescence in situ hybridization; CNVs: Copy number variations; ISCN: International system for human cytogenetic nomenclature; CT: Computerized tomography; IUGR: Intrauterine growth restriction; CGH: Comparative genomic hybridization; NAHR: Nonallelic homologous recombination; LCRs: Low-copy repeats; XLI: X-linked ichthyosis

## Competing interests

The authors declare that they have no competing interests.

## Authors' contributions

DL drafted the manuscript and participated in interpretation of the array results; MT and MB participated in providing clinical data and correcting the manuscript, especially on the clinical information; YF conceived of the study, and participated in its design and coordination, and edited the manuscript. All authors have read and approved the final manuscript.
